# Acquired Morgagni hernia following coronary artery bypass graft (CABG) with successful robotic repair of hernia

**DOI:** 10.1016/j.ijscr.2022.107164

**Published:** 2022-05-04

**Authors:** Steven A. Tamesis, Shahin Ayazi, Yoshihiro Komatsu, Meghan Allen, Blair A. Jobe

**Affiliations:** a Esophageal Institute, Department of Surgery, Allegheny Health Network, Pittsburgh, PA, United States; b Department of Surgery, Drexel University, Philadelphia, PA, United States

**Keywords:** Morgagni hernia, Diaphragmatic hernia, Robotic surgery, Mesh

## Abstract

**Introduction and importance:**

Morgagni hernia is an uncommon type of diaphragmatic hernia and commonly presents as a congenital disease. Acquired Morgagni hernias following open cardiac surgery are exceedingly rare and only reported in the pediatric population.

**Case presentation:**

The patient is a 70-year-old female who presented with complaints of shortness of breath and cough one year following a coronary artery bypass graft (CABG). A chest CT scan showed a large Morgagni type diaphragmatic hernia with herniated transverse colon occupying the anterior mediastinum as well as the right hemi-thorax. This hernia was successfully repaired using transabdominal robotic approach with complete resolution of patient's symptoms.

**Clinical discussion:**

This is the first reported case of acquired Morgagni type diaphragmatic hernia in an adult following open cardiac surgery. The potential etiologies for this hernia include distal extension of the median sternotomy and involvement of the anterior diaphragm, iatrogenic injury to the attenuated anterior diaphragm during pericardial window creation, or pericardial drain placement. Operative repair is the mainstay of treatment and is usually performed with a transabdominal approach since it is thought to be less challenging and allows for evaluation of the entire abdominal cavity. If primary repair cannot be achieved, then synthetic mesh may be needed to obtain a tension free and durable repair.

**Conclusion:**

We present a case of acquired Morgagni type diaphragmatic hernia in an adult following open cardiac surgery that was successfully repaired using a transabdominal robotic approach.

## Highlights

• Morgagni hernia is an uncommon type of diaphragmatic hernia and commonly presents as a congenital disease.
• Acquired Morgagni hernias following open cardiac surgery are exceedingly rare and only reported in pediatric population.
• Most Morgagni hernias can be approached thoracoscopically, laparoscopically, or robotically with reduction of hernia contents and diaphragmatic repair.
• If primary repair cannot be achieved, then synthetic mesh may be needed to obtain a tension free and durable repair.

## 1 Background

Morgagni hernia is an uncommon type of diaphragmatic hernia, it accounts for 2%–6% of all diaphragmatic hernias [1], [2]. Morgagni hernia most commonly presents as a congenital disease. This type of diaphragmatic hernia results from failure of complete migration of muscle fibers to cover a triangular space between the sternum and bilateral costal margins [1], [3]. Acquired Morgagni hernia on the other hand results from blunt or penetrating abdominal trauma, pregnancy, obesity, chronic constipation, and chronic cough [2], [4]. Acquired Morgagni hernias following open cardiac surgery are exceedingly rare and only reported in the pediatric population [2], [5], [6]. We present a case of acquired Morgagni hernia following an open cardiac surgery that was successful repaired using robotic approach. This work has been reported in line with the SCARE 2020 criteria [7].

## 2 Case presentation

**Fig. 1:**
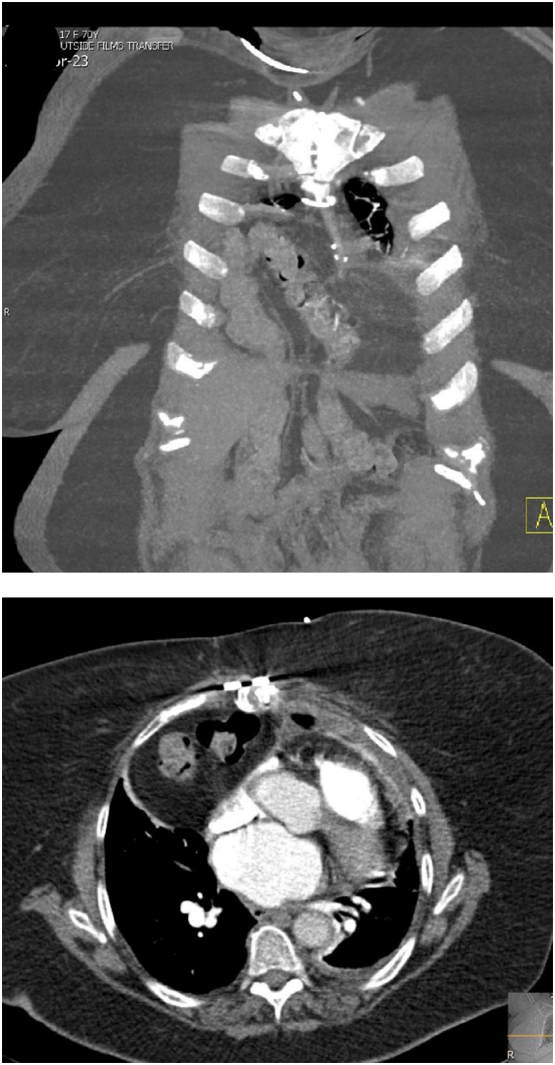
CT-scan of the chest and abdomen demonstrating a large anterior diaphragmatic hernia spanning to the anterior mediastinum and right hemithorax with herniated transverse colon and omentum.

**Fig. 2:**
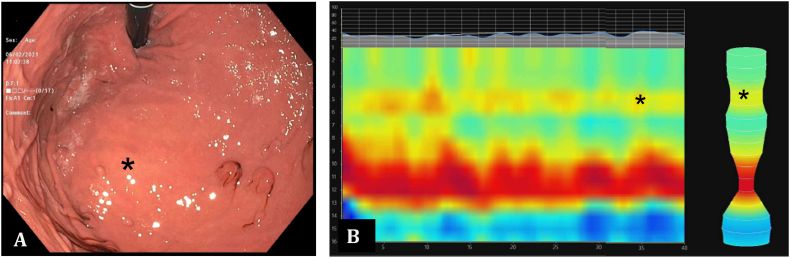
A) Endoscopic evidence of external compression in the fundus of the stomach (marked with *) corresponding with diaphragmatic hernia. B) The evidence of external compression in the distal esophagus (marked with *) on the FLIP topography panometry.

The patient is a 70-year-old female with a history of gastroesophageal reflux disease (GERD), hiatal hernia, coronary artery disease, congestive heart failure, hypertension, mitral valve prolapse, pre-diabetes, asthma, and obesity who presented with complaints of shortness of breath and cough. She underwent an urgent coronary artery bypass graft via median sternotomy one year prior to presentation. About 6 weeks after the procedure the patient started to develop progressively worsening shortness of breath with exertion and at rest. Her shortness of breath was so severe that she was not able to walk across a room without experiencing symptoms. This was associated with non-productive cough, and chest pain and pressure. She presented to her primary care physician and a CT scan of the chest was obtained. This imaging study showed a large anterior Morgagni type diaphragmatic hernia with herniated transverse colon occupying the anterior mediastinum as well as the right hemi-thorax (Fig. 1). The patient was then referred to our tertiary referral medical center in Pittsburgh for further evaluation and surgical management. The work-up consisted of an upper endoscopy with functional luminal image probe (FLIP) and Bravo pH monitoring as the patient was also complaining of significant reflux symptoms. Endoscopic examination showed a 2 cm sliding hiatal hernia and external compression along the distal esophagus and stomach which corresponded with the patient's known diaphragmatic hernia (Fig. 2-A). The external compression in the distal esophagus was also noticeable on the FLIP topography panometry (Fig. 2-B). Bravo pH monitoring showed increased distal esophageal acid exposure with DeMeester score of 49.4 and 45.8 for days 1 and 2 respectively. After discussion with the patient the decision was made to proceed with robotic repair of diaphragmatic hernia with mesh. Repair of her sliding hiatal hernia and anti-reflux surgery was also offered to the patient, but she elected to continue with medical management of her reflux disease.

## 3 Surgical procedure

**Fig. 3:**
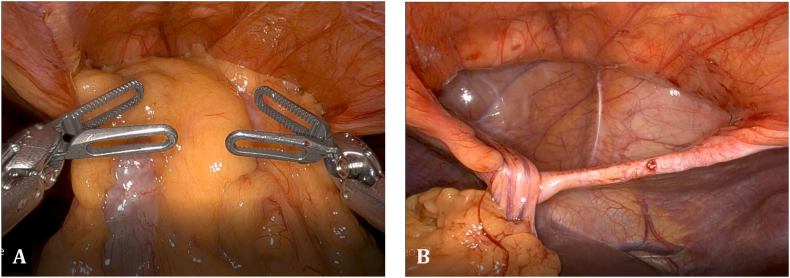
A) Large anterior diaphragmatic hernia with herniated transverse colon and omentum. B) Demonstration of a large hernia sac after reduction of transverse colon and omentum into the abdominal cavity.

**Fig. 4:**
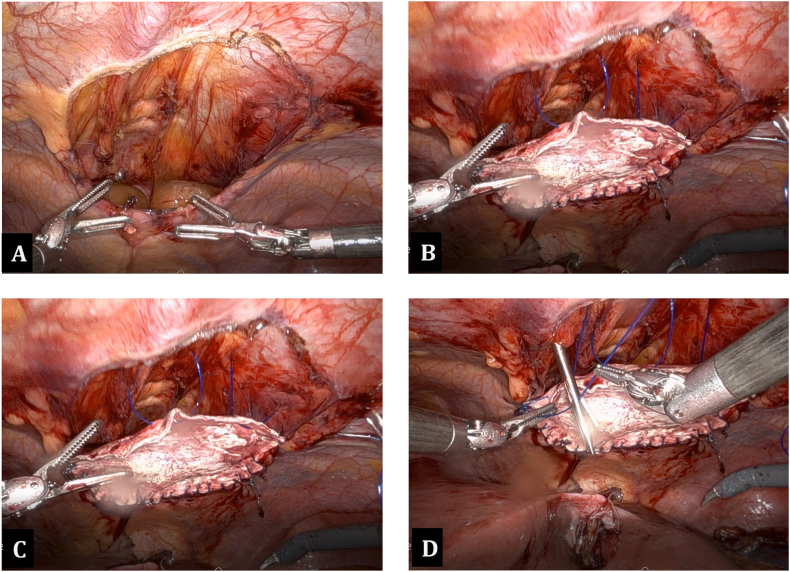
A) Anterior diaphragmatic hernia after full excision of the hernia sac. B) Lateral and posterior edge of the hernia was secured to the edge of the diaphragmatic defect using 2-0 Ethibond sutures and non-absorbable 0 v-lock sutures. C and D) Anterior edge of the anterior diaphragmatic defect fixed to the abdominal wall using non-absorbable 0 suture in a U stitch fashion, then brought out of the abdomen using suture passer device.

**Fig. 5:**
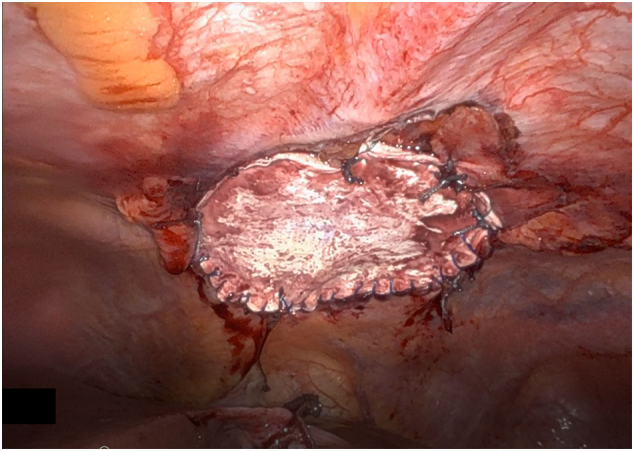
Anterior diaphragmatic defect completely closed with bridging Gore-Tex mesh.

**Fig. 6:**
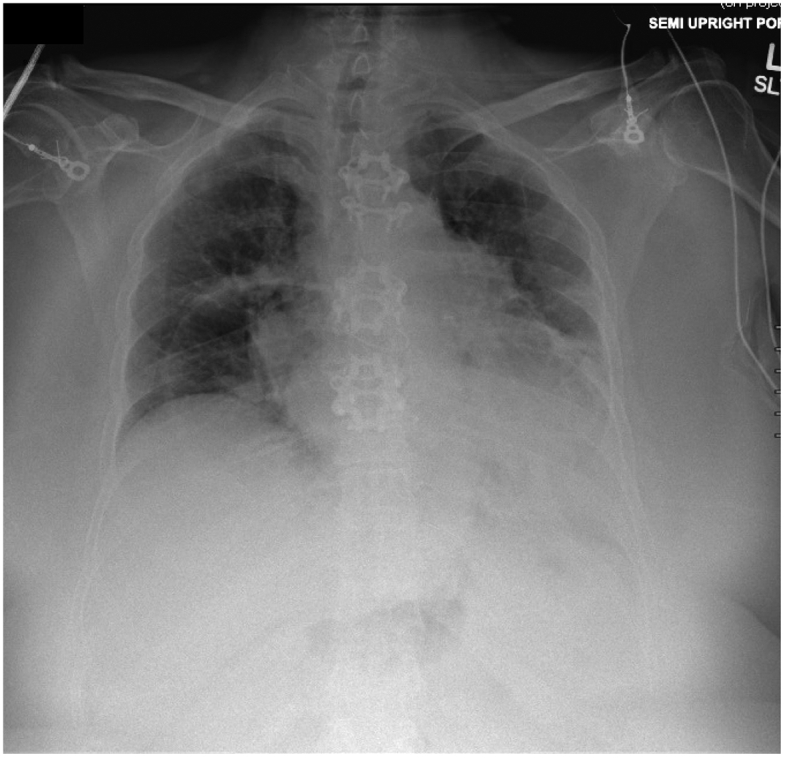
Chest X-ray obtained after surgery showing full resolution of the anterior diaphragmatic defect.

Upon entering the abdomen with a 5 mm 30-degree laparoscope there was note of a large anterior diaphragmatic hernia containing the transverse colon and omentum (Fig. 3-A). The viscera appeared to be viable. The remainder of the robotic ports was then placed and the DaVinci robot (Intuitive, Sunnyvale, CA, USA) was docked. The hernia contents were then carefully reduced using gentle downward traction (Fig. 3-B). Once the hernia contents were reduced the hernia sac was then completely dissected free from the defect using a combination of blunt and energy device dissection (Fig. 4-A). Once the hernia sac was completely excised the diaphragmatic defect was measured and found to be 7 cm × 8 cm in diameter. A piece of Gore-Tex mesh (W. L. Gore & Associates Inc., Newark, DE, USA) was cut to size and introduced into the abdomen. The mesh was appropriately positioned and then sutured into place along the two lateral edges and the midline posterior position using 2-0 Ethibond sutures (Ethicon, Somerville, NJ, USA). The lateral and posterior aspect of the mesh was then sown into place using multiple non-absorbable 0 V-loc suture (Covidien, Dublin, Ireland) (Fig. 4-B). The anterior defect was closed using multiple 0 non-absorbable suture in a U stitch fashion. A stab incision was then made on the abdominal wall along the edge of the mesh and the two ends of the suture were brought up through the abdominal wall at two different fascial points using a suture passer (Fig. 4-C and D). This was performed multiple times along the entire length of the anterior edge of the mesh. The anterior edge of the mesh was then further secured to the abdominal wall using 2-0 Ethibond sutures in a figure of 8 fashion. At the conclusion of the repair the entire hernia defect was completely closed (Fig. 5). Post operatively the patient did very well. She was discharged home on day 2 after surgery with improved shortness of breath. Chest X-ray post-operatively showed resolution of the diaphragmatic hernia (Fig. 6). The patient was seen in clinic recently on follow-up and was noted to be doing well with further improvement in her shortness of breath and activity tolerance.

## 4 Discussion

Diaphragmatic hernias can be classified as congenital or acquired. Of the congenital diaphragmatic hernias, the Bochdalek hernia accounts for nearly 80%, while the Morgagni hernia is far less common accounting for only 2–6% [1], [2], [8]. Morgagni hernia was first described in 1769 by the Italian anatomist Giovanni Battista Morgagni. These hernias are right sided in 90% of cases, left sided in 8%, and bilateral in 2% [8]. The most common herniated structure is the omentum (60%), followed by the colon, small bowel, stomach, and the liver [1], [5]. Acquired Morgagni hernias are even more rare and are due to direct penetrating trauma to the diaphragm resulting in herniation or the result of increase in the intra-abdominal pressure following blunt thoracoabdominal trauma, pregnancy, obesity, chronic constipation, or chronic cough [2], [4].

Presenting symptoms in the adult population are variable. A comprehensive literature review performed by Horton and colleagues showed that most patients present with chest and abdominal pain or pressure (37%) as well as pulmonary symptoms which consist of dyspnea, cough, and shortness of breath (36%). The remaining symptoms consist of obstruction (20%), dysphagia (3%), bleeding (1%) and GERD (1%) [9]. Variability with clinical symptomatology is also reflected in the pediatric population. In the pediatric population however, there is also an association with congenital anomalies of multiple organ systems that must be taken into consideration [10].

Diagnosis can be made with radiologic evaluation using chest x-ray, upper gastrointestinal contrast study, magnetic resonance imaging (MRI), and CT scan which can confirm the diagnosis in nearly all patients. Studies such as an upper endoscopy or endoluminal functional lumen imaging probe (EndoFLIP) technology in our patient provided additional information to support the diagnosis.

There are some reported cases of acquired Morgagni hernia following open cardiac surgery in children. Carballo et al. discuss the case of a 4-year-old female with a history of trisomy 21, ventricular septal defect (VSA), patent foramen ovale (PFO) and patent ductus arteriosus (PDA) who presented with acquired Morgagni hernia and pericardial hernia after open cardiac surgery. The patient presented with acutely incarcerated colon and small bowel into the right hemithorax, which was successfully reduced, and repaired using laparoscopic approach [2]. Another study by Bawazir et al. reported on 25 patients who presented from the period of 2005 to 2019 with Morgagni hernia. In this study 11 patients (44%) had congenital cardiac disease and presented after cardiac surgery via median sternotomy [5]. Yet another report by Panda and colleagues discussed the case of a 6-month-old girl who presented with an intra-pericardial diaphragmatic hernia following arterial switch operation with ventricular septal defect closure at 2 months of age. The patient presented with mild tachypnea. A chest x-ray was performed and showed a right paracardiac shadow. This was followed up with an echocardiogram which showed an intrapericardial mass compressing the right ventricle, resulting in early diastolic collapse and hemodynamic compromise. Chest CT-scan was significant for what was thought to be a paracardiac organized thrombus. On redo median sternotomy this mass was discovered to be the left lobe of the liver and omentum herniated through a 2 cm × 3 cm anteromedial diaphragmatic defect [6].

In our report, the patient is a 70-year-old female with no history of congenital Morgagni hernia, no history of penetrating or blunt thoracoabdominal trauma, and without risk factors for the development of acquired Morgagni hernia aside from obesity. Furthermore, the imaging prior to her CABG did not show any evidence of diaphragmatic hernia. To the best of our knowledge, this is the first report of Morgagni type of diaphragmatic hernia in an adult following cardiac surgery.

There are two plausible explanations for the development of an acquired Morgagni hernia following cardiac surgery. The first is distal extension of the median sternotomy and involvement of the anterior diaphragm resulting in a direct communication between the thoracic and abdominal cavities. Panda et al. discuss that this occurs due inferior extension of the median sternotomy to facilitate exposure of the heart and that inadvertent breach of the peritoneum results in late herniation of the visceral contents into the pericardial cavity. This can be avoided by limiting the inferior extent of the sternotomy and extending this preferentially in a T-shaped fashion above the diaphragm if more exposure is required [6]. The second explanation is that following cardiac surgery drains are commonly placed in the substernal area. Pericardial drains are also placed in this area during pericardial window creation. Placement of these drains can lead to injury to the anterior diaphragm and may result in poor tissue healing which can contribute to the development of acquired Morgagni hernia [5]. Review of the anatomy of the diaphragm shows that the sternal attachment of the diaphragm consists of two small muscle bundles that attach to the xiphoid process. Between these two muscle bundles, there is an irregular opening known as Larrey's fissure. Laterally to the muscle bundles are two interstices (Morgagni foramen) covered by pleura and the adjacent pericardium [11], [12]. These areas are potential weakness points within the diaphragm and injury to these areas may lead to the development of acquired Morgagni hernia.

Operative repair of acquired Morgagni hernia can be approached through the abdomen or the chest. The abdominal approach is commonly preferred; it is thought to be less challenging and provides ability to examine the entire abdominal cavity and the viscera, in addition allows evaluation of the contralateral diaphragm for additional defects [12]. Nakashima and colleagues contend that in an adult patient the thoracoscopic approach may offer the advantage of safer adhesiolysis [13]. The chronic nature of the disease in adult population often results in more significant adhesions compared to the pediatric population where adhesions are usually not very significant. Thoracoscopic approach is also preferred by some authors in morbidly obese patients as provides easier access to the hernia [14].

Excision of the hernia sac is also a matter of debate. Excision of the hernia sac is considered a surgical principle which is thought to decrease the risk of hernia recurrence [14]. Some authors have argued that hernia sac can be densely adherent to the intrathoracic structures which may be injured during the excision [5], [12], [14]. However, Kao and colleagues reported a series of 15 patients with congenital Morgagni hernia that were all repaired via transabdominal approach with complete excision of sac. They report no post-operative complications in their series relating to hernia sac excision [15].

Once the hernia contents are reduced and the defect is well-defined, the diaphragmatic repair can be achieved primarily, with mesh interposition, or with mesh reinforcement. The choice to use mesh or to repair the defect primarily depends largely on the size of the defect with Thoman et al. recommending prosthetic repair for defects with surface area greater than 20–30 cm^2^ [16].

## 5 Conclusion

We reported the first case of acquired Morgagni type diaphragmatic hernia in an adult following an open cardiac surgery. These hernias are most likely caused by iatrogenic injury to the attenuated anterior diaphragm during pericardial window creation, pericardial drain, chest tube placement, and distal extension of median sternotomy. Surgical repair remains the mainstay of treatment for Morgagni hernia due to the high risk of strangulation of herniated contents. There is no standardized technique for repair, however, most Morgagni hernias can be approached thoracoscopically, laparoscopically, or robotically with reduction of hernia contents and diaphragmatic repair. If primary repair cannot be achieved, then synthetic mesh may be needed to obtain a tension free and durable repair.

## Consent

Yes.

## Provenance of peer review

Not commissioned, externally peer-reviewed.

## Ethical approval

Yes.

## Funding

N/A.

## Guarantor

Shahin Ayazi, MD.

## Research registration number

None.

## CRediT authorship contribution statement

Equal contribution by all authors.

## Declaration of competing interest

None.
